# Development and pilot of an online, personalized risk assessment tool for a breast cancer precision medicine trial

**DOI:** 10.1038/s41523-021-00288-8

**Published:** 2021-06-17

**Authors:** Holly Keane, Yash S. Huilgol, Yiwey Shieh, Jeffrey A. Tice, Jeff Belkora, Karen Sepucha, W. Patrick Shibley, Tianyi Wang, Mandy Che, Deborah Goodman, Elissa Ozanne, Allison Stover Fiscalini, Laura J. Esserman

**Affiliations:** 1 Department of Surgery, University of California, San Francisco, San Francisco, CA USA; 2 Peter MacCallum Cancer Centre, Melbourne, Australia; 3 School of Medicine, University of California, San Francisco, San Francisco, CA USA; 4 Joint Medical Program, School of Public Health, University of California, Berkeley, Berkeley, CA USA; 5 Institute for Health Policy Studies, University of California, San Francisco, San Francisco, CA USA; 6 Health Decision Sciences Center, Massachusetts General Hospital, Boston, MA USA; 7 Department of Epidemiology, University of California, Irvine, Irvine, CA USA; 8 Department of Population Health Sciences, University of Utah School of Medicine, Salt Lake City, UT USA

**Keywords:** Cancer prevention, Cancer screening, Medical research, Population screening, Epidemiology

## Abstract

Breast cancer risk reduction has been validated by large-scale clinical trials, but uptake remains low. A risk communication tool could provide personalized risk-reduction information for high-risk women. A low-literacy-friendly, visual, and personalized tool was designed as part of the Women Informed to Screen Depending On Measures of risk (WISDOM) study. The tool integrates genetic, polygenic, and lifestyle factors, and quantifies the risk-reduction from undertaking medication and lifestyle interventions. The development and design process utilized feedback from clinicians, decision-making scientists, software engineers, and patient advocates. We piloted the tool with 17 study participants, collecting quantitative and qualitative feedback. Overall, participants felt they better understood their personalized breast cancer risk, were motivated to reduce their risk, and considered lifestyle interventions. The tool will be used to evaluate whether risk-based screening leads to more informed decisions and higher uptake of risk-reduction interventions among those most likely to benefit.

## Introduction

The development of risk models has not led to the uptake of risk-reducing interventions, such as endocrine risk-reduction^1^. Large-scale clinical trials of selective estrogen receptor modulators and aromatase inhibitors show benefit in high-risk women for reducing both invasive and non-invasive breast cancer incidence^2–9^. However, initial studies of quality of life have significantly underrepresented the significant side-effects of medications, which impact a patient’s willingness to consider risk-reduction^3,5,6,10^. These side-effects include menopausal symptoms, thromboembolism, and endometrial cancer risk; recent literature suggests these possibly correlate with dosage^11–13^. In addition to risk-reducing medications, several studies have demonstrated that lifestyle interventions also may reduce the risk of breast cancer^14–19^.

Risk-reduction strategies are only useful if women are aware that they are estimated to have high risk and benefits outweigh the risk of side-effects^20,21^. The US Preventive Services Task Force (USPSTF) guidelines recommend the use of clinical risk models to assess women of high risk^22^. Early models were first created by Gail and used in the Breast Cancer Risk Assessment Tool^23,24^. Since then, models such as the Tyrer-Cuzick, Breast and Ovarian Analysis of Disease Incidence and Carrier Estimation Algorithm (BOADICEA), and the Breast Cancer Surveillance Consortium (BCSC) have enhanced prior models by incorporating mammographic density for their 5- and 10-year risk projections^19,25,26^. Model modifications integrating genetic risk have been put into practice^27–29^. The BCSC model has been validated in a diverse population of over 1 million women across the United States.

In a review of available breast cancer risk assessment tools, we found the following barriers to informed adoption of endocrine risk reduction:

First, it is difficult to understand personal risk and whether the risk of side-effects outweigh the benefits. Prior risk assessment tools have attempted to make breast cancer risk information more readily accessible for primary care physicians to use online^30,31^. The development of these frameworks has been beneficial, but these tools are often provider facing, and do not improve the ability of patients to understand the information.

Second, most online, patient-facing tools do not incorporate an individual’s genetic testing information that would be needed to personalize risk information^32,33^. As advances in next-generation sequencing (NGS) became cheaper in the precision medicine era, they accelerated the access to and use of broader germline gene panels to assess individual risk. Tailoring risk-reduction based solely on standardized factors included in the Gail, Tyrer-Cuzick, and BCSC models was not as comprehensive. However, as the use of NGS increases, so does the need for risk assessment tools to explain and interpret personal genetic and polygenic risk in a meaningful way for patients.

Third, risk assessment tools need to make risk and risk-reduction information readily understandable. A study identified that 63% of patient educational materials randomly sampled from MedlinePlus/National Library of Medicine were above the reading level of an average United States resident (eighth-grade equivalent or below, based on the Flesch-Kincaid scale)^34–37^. This suggests that risk information would not be easily understood by those with lower health literacy. Decision scientists suggest the use of natural frequencies and absolute risk help patients understand medical risk^38,39^.

Members of our research team (E.O., L.J.E.) had previously developed a risk assessment tool to guide and inform high-risk consultations in breast cancer^30,40^. The risk assessment tool described in this paper aims to improve upon this previous tool^30^. We will test the efficacy of the tool in the Women Informed to Screen Depending On Measures of risk (WISDOM) study, a precision medicine trial administered by the Athena Breast Health Network^28^.

## Results

### Finalized risk assessment tool

The prior version of the computer-based risk assessment tool inspired the development of five primary pages^30^. Each page allowed the participant to build an increasingly nuanced understanding of their risk and ways of mitigating their risk. Following iterative initial user testing with the multidisciplinary advisory board and visual/software changes to incorporate their feedback, the five primary pages developed were:

1. *My risk snapshot*: Reviews risk calculation inputs, as provided by participants in a WISDOM Study Breast Health Questionnaire, that are used to calculate both the Gail and BCSC scores. For each input, the page provides the user-submitted information for age, race, family history, breast biopsy history, and breast density. The page also explains the WISDOM Study’s personalized screening recommendation, risk calculations, and polygenic risk score.
2. *My risk report*: Uses both natural frequencies and icon array diagrams to provide the risk of developing breast cancer versus an average woman of the same age and race/ethnicity. The participant can toggle between 5-year, 10-year, and lifetime risks, or compare all of them simultaneously (Fig. 1).

**Fig. 1:**
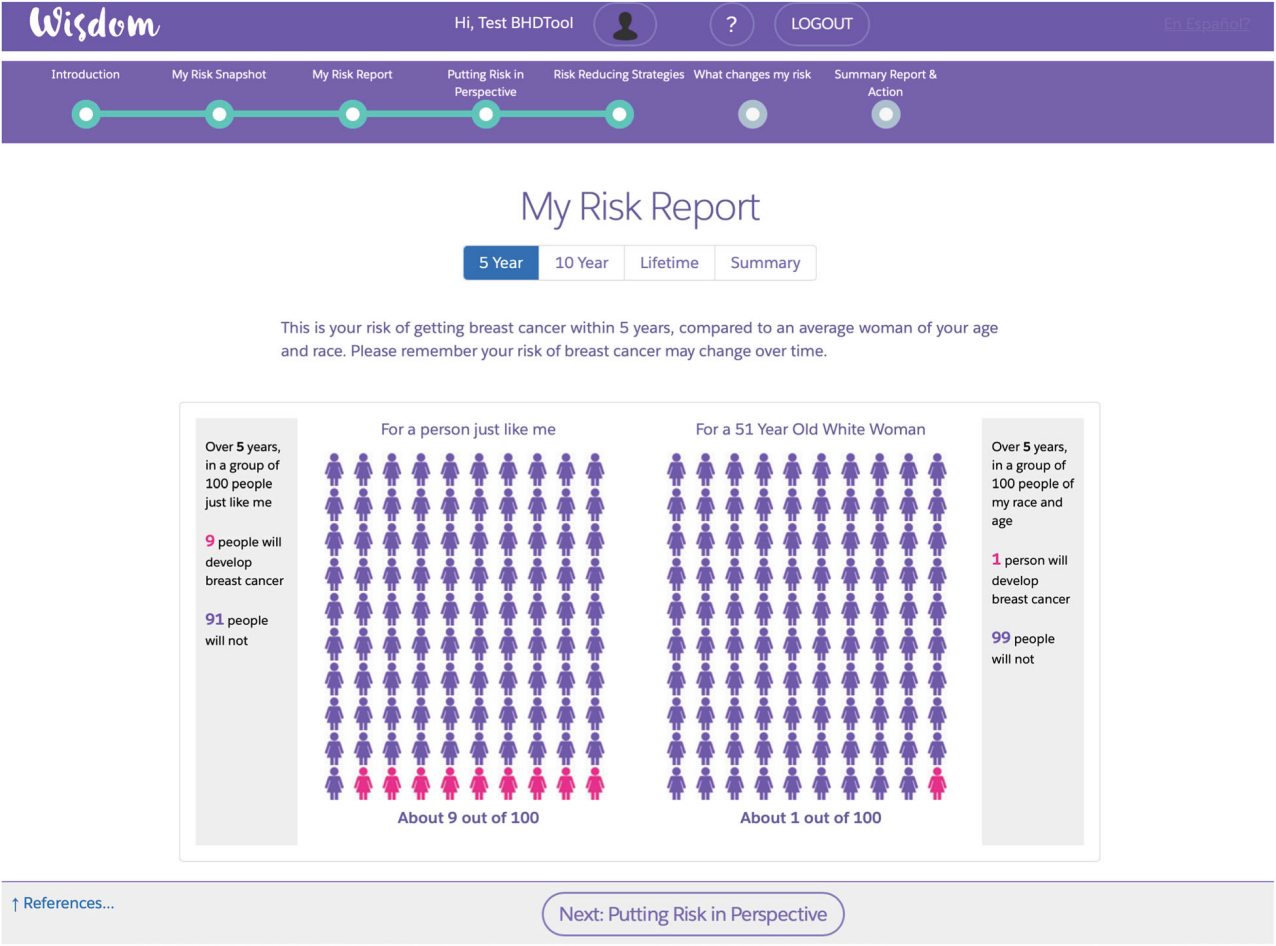
My risk report. This primary page compares a participant’s five-year, ten-year, and lifetime (estimated to age 90) risk of developing breast cancer in comparison to an average woman of the same age and race/ethnicity.
3. *Putting risk in perspective*: Uses natural frequencies and diagrams to contrast a woman’s risk of developing breast cancer over 10 years in the context of other common morbidities such as heart attack and stroke (using SEER program data estimates).
4. *Risk-reducing strategies*: Provides both medication, lifestyle, and surgical options for women to reduce their risk of breast cancer. If rigorous, peer-reviewed information is available, we also provide the estimated absolute risk-reduction from performing that intervention. Each medication and lifestyle intervention link to secondary pages (Fig. 2) that include information on side-effects or benefits. Each strategy is personalized based on a woman’s menopausal status, age, or Breast Health Questionnaire inputs.

**Fig. 2:**
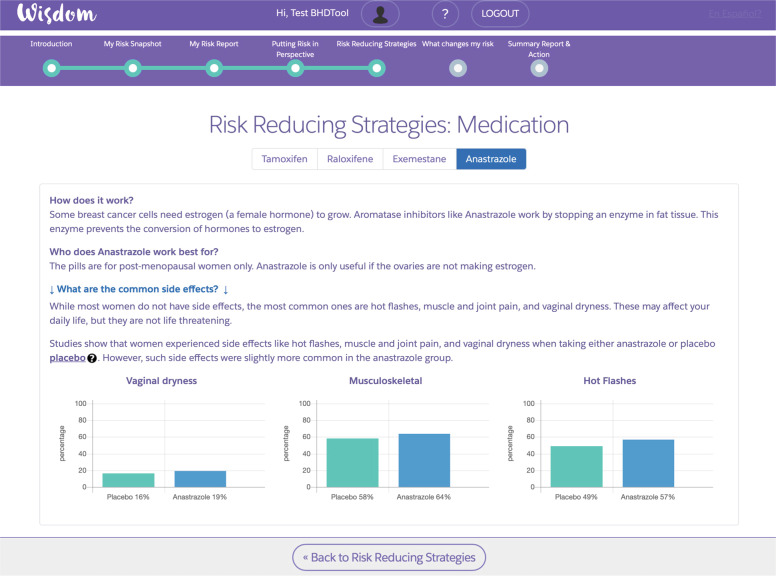
Detailed page on medication. This is an example of a secondary page that includes detailed information on side-effects and benefits. Each strategy is personalized based on a participant’s menopausal status, age, or questionnaire inputs. Participants can toggle through different aromatase inhibitors and selective estrogen receptor modulators (SERMs) that might be appropriate for them.
5. *Exploring what changes my risk*: The tool summarizes in a graphical form the different risk-reducing strategies presented on the “Risk-reducing strategies” page. Each absolute risk score is modified based on the estimated change to breast cancer risk (Fig. 3).

**Fig. 3:**
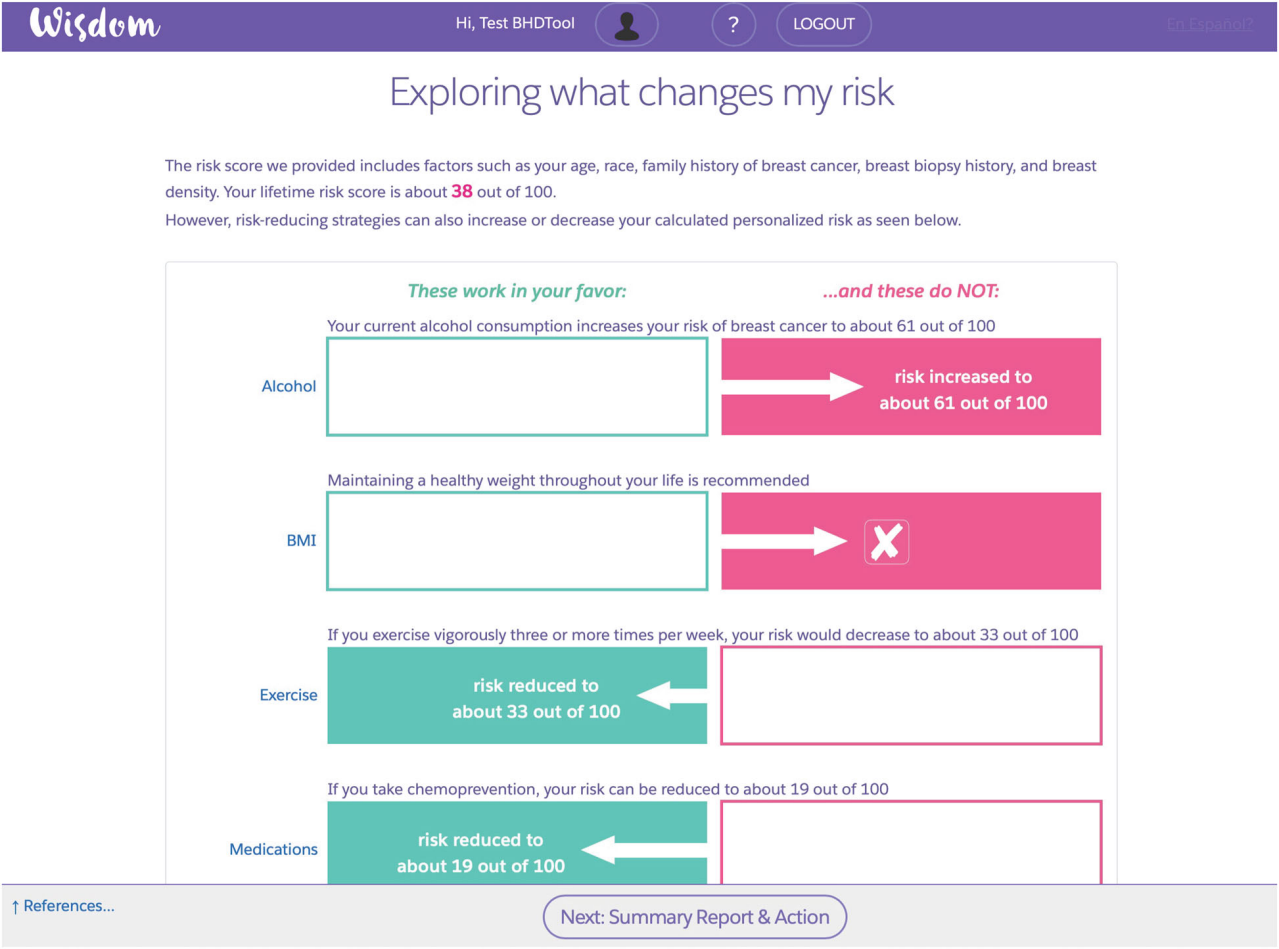
Exploring what changes my risk. This is an example of a primary page summarizing how different risk-reducing strategies impact a participant’s absolute breast cancer risk.

Participants were also given the option to view more information via hyperlinks given on primary pages. A PDF version of an individualized risk information/report was accessible on the final summary page of the tool. Peer-reviewed articles used to develop each primary page were also compiled. All references were linked and available on secondary landing pages.

Reading ease and grade level^36^ of the pages are shown in Table 1. The grade level was 8 and below for all pages. Reading ease varied from a low of 53.8% to a high of 90.2%.

**Table 1:** Page description and calculated readability.

Primary pages	Description	Reading ease	Grade level
Introduction	Introduces tool and navigation schemes	78.2	6.2
My risk snapshot	Lists risk factors used in Gail or BCSC risk calculation and why they are important.	59.7	8.1
My risk report	Provides 5-year, 10-year, and lifetime risk of developing breast cancer vs an average woman of the same age and race.	85.6	4.3
Putting risk in perspective	Places risk in the context of other common morbidities.	90.8	3.6
Risk-reducing strategies	Suggests medicine and lifestyle options for risk-reduction based on risk factors, menopausal status, and age.	59.4	8.8
Exploring what changes my risk	Estimates new absolute risk based on intervention.	53.8	8.8
What’s next?	Provides 4-question Breast Health Risk Assessment Tool survey and option to export results for primary care physicians.	78.1	4.0

The primary page and descriptions are provided along with the corresponded calculated readability and grade level. Flesch-Kincaid reading ease and grade level are based on Readability Test Tool, developed by WebFX^36^. An average United States resident has an eighth-grade reading level. Secondary pages that elaborate on details reported in peer-reviewed journals may have higher-grade levels.

### Pilot study participants

A convenience sample of 20 elevated risk WISDOM Study participants was identified in the personalized arm without a breast cancer mutation (namely BRCA1, BCRA2, TP53, PTEN, STK11, CDH1, ATM, PALB2, CHEK2). These women were all classified as elevated risk, in the top 2.5% of BCSC score by age. A total of 17 women responded to the invitation to participate in the walk-through with a genetic counselor. Demographic data were obtained from self-reported responses in the WISDOM Breast Health Questionnaire administered at baseline enrollment and re-assessed in follow-up years (Table 2). Women who participated in the pilot study were mostly white, college-educated or higher, and between ages 40 and 74.

**Table 2:** Demographic distribution of pilot participants.

Total participants		*N* = 17
Age	40–49	5
	50–59	5
	60–69	5
	70–79	2
Education	High School Graduate or less	0
	College Graduate or More	17
Race/Ethnicity	White	16
	Black or African American	0
	Asian	0
	Hispanic, Latino, or Spanish Origin	1
	Other	0

Describes the primary demographic distribution of pilot study participants (*N* = 17).

### Quantitative pilot results

Of the 17 participants who were given the walk-through of the tool with a genetic counselor, 14 participants agreed to respond to the quantitative survey (Table 3). All 14 participants indicated that they had a better understanding of their breast cancer risk after using the personalized risk assessment tool. 13 of the 14 participants indicated that the tool helped facilitate their understanding of risk; 6 participants indicated that the tool was “Extremely helpful” while 7 participants indicated that it was “Very helpful” for them to understand their breast cancer risk.

**Table 3:** Quantitative feedback survey responses.

	Participants surveyed (*N* = 14)
Q1: *Was the visual decision guide helpful for you to understand your breast cancer risk?*	
Not at all	0
Somewhat helpful	1
Very helpful	7
Extremely helpful	6
Q2: *Do you better understand your chance of developing breast cancer after using this visual decision guide?*	
Not at all	0
Somewhat	1
Yes	13
Q3: *What are you considering doing to reduce your chances of developing breast cancer? Please check all that apply:*	
Nothing at this time	3
Risk-reducing medication	6
Decreasing alcohol intake	3
Increasing exercise	6
Losing weight	6
Surgical options	1
Q4: *How motivated are you to reduce your chance of developing breast cancer?*	
Not at all	0
Somewhat motivated	3
Very motivated	6
Extremely motivated	4

Findings from the quantitative feedback survey responses (*N* = 14). Question 3 was multiple-select, so total responses are greater than the number of participants who completed the questionnaire.

Of the 14 participants, 10 indicated that they were “Extremely motivated” or “Very motivated” to reduce their breast cancer risk. Of those, 6 were interested in taking risk-reducing medication. 10 women indicated they were considering lifestyle modifications to reduce their risk, such as exercising, decreasing alcohol intake, and reducing their body mass index. 1 participant indicated that she is considering surgical options.

### Qualitative pilot results

From the pool of 14 participants who gave quantitative feedback, 10 participants agreed to provide qualitative feedback. Similar to the responses in the quantitative survey, most women report benefiting from the tool’s visual content.

Nine out of the 10 interviewed women had positive comments about the visuals. Five women specifically mentioned that they were visual learners with comments including:

“Just [talking] on the phone would not have had as big of an impact… I see the visuals in my mind, as I think back”.

“I’m a visual learner and for others who are, having a knowledgeable professional walk you through the infographics simplified the understanding process”.

Four women emphasized how the visuals accompanying the physician’s verbal explanations improved their knowledge retention, with one stating that she liked the process of the doctor guiding her through the tool while being able to visually see the tool being used. Two women stated that they could not “un-see” what was shown in the tool, so “seeing it [the visuals] was a motivating factor.”

Four women particularly liked the My Risk Report page (Fig. 1), which displayed the participant’s specific risk of developing breast cancer. One participant said in detail:

“[I] like the side-by-side comparison of me versus an average 40-year-old. That helped make a connection and gives you a scale visually. For people for whom numbers are abstract…that stark reminder is good. Seeing the picture makes it click.”

Five of the interviewed women learned more about their risk, with two realizing that their risk was higher than they previously thought. One of them stated:

“I never gave too much thought to my personal risk… no one in my family has breast cancer…presumed my risk was low. I had no idea that breast density was a risk factor and the consequences of that.”

Three participants were not aware of risk-reduction medications before their risk assessment consultation. One stated that:

“I was completely unaware about medication-related therapy. I had no idea that there were prescription medications to reduce your risk. I am not sure if I’m particularly interested in starting at this moment, but I would look into finding out more about the risks and side-effects for the benefits.”

Five participants plan to speak with their providers about risk-reduction medications. One participant discussed learning about them for the first time:

“[I] had no idea that there were medications one could take to reduce risk…Why do women not know more about these options? I am great about my health and this idea that there is this option out there…that doesn’t seem very hard to do…not as severe as you know, having a [prophylactic] mastectomy.”

Negative feedback was also collected, with a majority focusing on clarity of how risk models calculated, their inputs, and suggested risk-reduction interventions. Interviewees expressed frustration with understanding the tool’s use of various risk model inputs and risk score calculations:

“I thought it was weird that there was no good explanation for why having normal, benign breast biopsy puts you at higher risk. That wasn’t logical to me.”

“I am disappointed that I went through a great deal of trouble [to complete the surveys]. I sent a lot of information [to the WISDOM Study] to find that it wasn’t accurately applied to determine my risk.”

“It didn’t take into consideration that my mother developed breast cancer at age 80.”

One participant expressed disappointment with the risk-reduction options mentioned in the tool:

“[I was] disappointed that there weren’t any options other than mammograms and MRIs and some lifestyle changes and medications offered as prevention.”

## Discussion

The development and pilot of the Women Informed to Screen Depending on Measures of risk (WISDOM) study risk assessment tool improves upon a previously developed risk assessment tool from 2014^40^. The tool introduces a woman to her predicted risk of breast cancer and contextualizes among other women and common morbidities. The tool builds on and accommodates the evolving field of risk assessment and risk reduction, which includes risk modified by genetic/polygenic information. The tool is also interactive and patient-facing, allowing women to access as much detail as they wish with a facilitator. Finally, we designed the tool specifically to be low-literacy-friendly at an eighth-grade reading level or lower.

Our pilot study suggests that a review of risk using this tool with a genetic counselor could be used to inform and motivate women who either previously believed their risk was low or unaware of risk-reduction medications to seek further professional guidance. However, in response to pilot feedback from participants requesting more detail on factors contributing to their risk scores, we revised sections discussing why certain inputs are included. One participant identified one of the key problems with biopsy history as a risk factor, namely that the Gail model was developed before the screening era. Today, the Gail model overestimates risk due to the increased number of biopsies of non-palpable masses identified by a mammogram. We also explicitly mentioned how participant responses from the annual WISDOM Study health questionnaires are used as inputs in risk score calculations.

Overall, participants in the pilot study found that incorporating personalized genetic data and readability metrics in the tool’s content useful in understanding their breast cancer risk and risk-reduction options. Ten of fourteen participants indicated that they were “very motivated” and “extremely motivated” to reduce their risk. Participants also said that they would consider medication and lifestyle modifications, though more participants were motivated to pursue lifestyle modifications than risk-reducing medication. This could be due to the lower barrier in beginning lifestyle changes and consideration of side-effects, as discussed elsewhere^11^.

Follow-up will be necessary to determine participant statements translate into action. As part of our follow-up, we will find out whether they discuss these preventive options with their primary care physicians. The pilot suggests that the tool can improve a woman’s knowledge of risk-reducing strategies and thereby lead to informed breast health choices. It is clear that even those who consider themselves well-informed, even self-proclaimed health enthusiasts, are not aware of risk-reducing medications and their potential benefits, especially for high-risk women. The research milieu of breast cancer decision-making tools has been focused on informed choice, and the contribution of literacy requirements and graphics seem to be in line with building on prior efforts^41^.

Our study has several limitations, which could be addressed in further studies. First, our pilot used a small sample size of mostly white, well-educated participants, which is not representative of the general breast cancer screening population. Furthermore, convenience sampling is prone to selection bias, because we contacted participants who had completed their annual questionnaire when the tool was completed. To make the study findings more representative of the general population, a larger study sample with diversity across education, age, and race is essential^42^. Second, the tool was designed and analyzed for low reading grade level, but we have not analyzed the tool for health literacy in terms of numeracy. Efforts are currently underway as part of a validation survey to include a more diverse study population and learn more about the numeracy requirements to using the tool. Third, the pilot study required a walk-through of the tool with a genetic counselor. Therefore, navigation and comprehension could have been improved by the genetic counselor clarifying information. Finally, since data are populated with a patient’s specific risk information as collected through the WISDOM Study, the risk assessment tool is only accessible for study participants at this time.

Based on encouraging feedback from the pilot study, we are collecting additional follow-up data on a larger cohort of elevated risk study participants^43^. We are motivated to recruit women of greater diversity in education, race, and ethnicity, to further evaluate the tool’s role in risk knowledge and risk-reduction strategies. The tool is being actively used with participants in the top 2.5% of risk by age as part of a consult with WISDOM Study Breast Health Specialists or genetic counselors, as this group was identified as a population where we would be most likely to improve the uptake of risk-reduction strategies^44^. Extending upon the current pilot data collected, we will evaluate the participant’s concrete actions after exposure to the risk assessment tool as part of the consultation.

However, it will also be important to assess if the tool is effective for participants navigating the tool independently, as the tool will be released to a wider WISDOM Study sample. Throughout this process, additional modifications are likely necessary to improve the tool’s usability and conveyance of risk information. The study team is also identifying methods of sharing these resources with participants’ primary care physicians. We hope to automate the communication from WISDOM Study risk assessment tool to the participant’s primary care provider.

As the tool standardizes the presentation of risk information, it will be used to test the fourth aim of the WISDOM Study, if (1) Personalized risk-based screening promotes endocrine risk-reduction uptake. (2) Use of the risk assessment tool results in more active participation in risk reduction in the personalized screening arm compared to the annual screening arm. After validation in the WISDOM study, we hope to generalize the tool and enable it to be integrated into healthcare consultations as part of primary care.

## Methods

### Development process

The WISDOM Study was approved by the University of California, San Francisco (UCSF) Institutional Review Board, which included the development and pilot of our risk assessment tool. Informed consent was obtained from all human participants. The Women Informed to Screen Depending on Measures of risk (WISDOM) study is registered on ClinicalTrials.gov as NCT02620852.

**Fig. 4:**
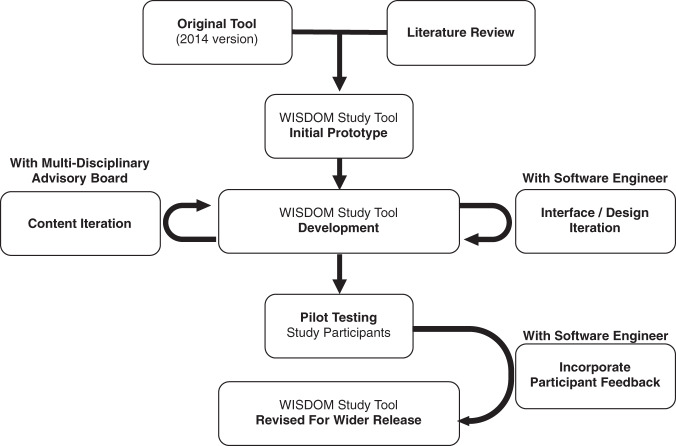
Development and pilot process for the WISDOM study risk assessment tool. Schematic describing the development and pilot process, which began with a review of the literature and the 2014 version of the tool. The content was developed with a multidisciplinary advisory board. Interface and design were iterated with a software engineer, following feedback from the advisory board and study participants.

The primary focus was three-fold: to incorporate personalized risk assessments; to create a patient-facing web-based software application; to make the risk assessment tool accessible to a low-literacy audience. Figure 4 illustrates the development process for the risk assessment tool.

First, authors HK and YSH reviewed the 2014 version of the tool and assessed the breast cancer risk-reduction literature to identify the highest level of evidence regarding modifiable and non-modifiable risk factors for breast cancer^30^. The technology and design phase involved close work with a software engineer and authors HK and YH designing a prototype for the digital Salesforce platform. The requirements of the prototype included the ability to interface with the existing study platform used by the WISDOM Study. This is because the WISDOM Study participant risk assessment, risk calculation, and survey entries are all housed on the Salesforce-based WISDOM Study authenticated portal to protect patient privacy. Another requirement was to design the tool to be accessible by computer, tablet, and smartphone web browsers.

### Multidisciplinary advisory group

To review its design, the authors convened an advisory group was composed of 3 physicians, 4 breast cancer researchers, 4 patient advocates, 2 genetic counselors, 3 behavioral and decision scientists, 1 user interface and user experience developer, 1 software engineer, and 1 programmer. The group met eight times during the calendar years 2017–18, partially in-person and otherwise virtually. Authors H.K. and Y.H. elicited and summarized the group’s input on the content and presentation of risk communication. Per their recommendations, the tool incorporated evidence-based principles for risk communication described in the literature.

### Assessing tool readability

We used the Readability Test Tool from WebFX to determine the Flesch-Kincaid Reading Ease and Grade Level^36^. Our goal was to ensure the instructions and curated content of primary pages were at an eighth-grade reading level or below.

### Participant risk estimates

We used participant survey data and mammographic density data from the WISDOM Study intake questionnaire to determine a woman’s 5- and 10-year risk of developing breast cancer. 5- and 10-year risk calculations used the Breast Cancer Surveillance Consortium (BCSC) score. We additionally used a patient’s genetic testing results (including intermediate-high penetrance genes and single nucleotide polymorphisms) to estimate participant’s risk of developing breast cancer via a polygenic risk score. This model has been published previously^45^.

### Pilot study process

We designed a pilot study intended to obtain feedback on the tool from participants evaluated to be high-risk, non-gene mutation carriers. The purpose of the pilot study was to improve upon the tool before roll-out to a larger study sample, and assess the conveyance of personal risk knowledge and motivating preventative actions immediately after consultation with a genetic counselor.

The participants were identified by the following procedure. First, 20 high-risk participants were identified to be at elevated risk for developing breast cancer and without a known mutation associated with breast cancer. These participants had completed their annual WISDOM Study Breast Health Questionnaire in January 2019, before the tool was to be piloted. Then, participants were contacted by the study team by email and offered the opportunity to participate in a walk-through of their risk assessment with the tool by a genetic counselor.

Those who accepted the invitation were guided systematically through each of the pages by a genetic counselor (DG) using a virtual screen sharing function in the Zoom platform. The genetic counselor was able to refer to the secondary pages and also respond to participant questions during the session. After the guided consultation, members of the study team asked the participant if they were willing to provide quantitative and qualitative feedback. The purpose of the four-question quantitative survey (see Supplementary Note 1) was to assess the utility of the tool in informing women about their breast cancer risk along with their motivation to undertake any of the risk-reducing strategies after learning about their risk. If participants were willing, follow-up phone interviews for qualitative feedback were also conducted by authors Y.S.H., T.W., and M.C. until thematic saturation using a semi-structured interview guide (see Supplementary Note 2).

### Reporting summary

Further information on research design is available in the Nature Research Reporting Summary linked to this article.

## Supplementary information

Supplementary Information

Reporting Summary

## Data Availability

The data generated and analyzed during this study are described in the following data record: 10.6084/m9.figshare.14546058^46^. The personalized risk assessment tool and the data supporting the validity of this tool are available on request from the corresponding author, Laura J. Esserman (Laura.Esserman@ucsf.edu). The tool is available for non-commercial use only and the data are not publicly available since the WISDOM Study portal requires participant authentication and registration.

## Code availability

Readability Test Tool from WebFX was used to determine the Flesch-Kincaid Reading Ease and Grade Level. This tool is publicly available online^36^.
