# Supplementary material for: Development and pilot of an online, personalized risk assessment tool for a breast cancer precision medicine trial
Source: NPJ Breast Cancer. 2021 Jun 17;7:78. doi: 10.1038/s41523-021-00288-8 (PMC8211836; doi:10.1038/s41523-021-00288-8)
Supplement: Supplementary file 1 — Supplementary Information [file 41523_2021_288_MOESM1_ESM.pdf]

Article

Development and Pilot of an Online, Personalized Risk Assessment Tool for a Breast Cancer Precision Medicine Trial

Keane H, Huilgol YS, et al.

Supplementary Information:

Supplementary Note 1. Quantitative Feedback Survey Questions

Supplementary Note 2. Qualitative Feedback Interview Guide

Supplementary Note 1: Quantitative Feedback Survey Questions

1. Was the visual risk assessment guide helpful for you to understand your breast cancer risk?
    a. Not at all
    b. Somewhat helpful
    c. Very helpful
    d. Extremely helpful
2. Do you better understand your chance of developing breast cancer after using this visual risk assessment tool?
    a. Not at all
    b. Somewhat
    c. Yes
3. What are you considering doing to reduce your chances of developing breast cancer? Please check all that apply:
    a. Nothing at this time
    b. Risk reducing medication (or *chemoprevention*)
    c. Decreasing alcohol intake
    d. Increasing exercise
    e. Losing weight
    f. Surgical options
4. How motivated are you to reduce your chance of developing breast cancer?
    a. Not at all
    b. Somewhat motivated
    c. Very motivated
    d. Extremely motivated

Supplementary Note 2: Qualitative Feedback Interview Guide

Reconfirm consent

1. You may have noticed that the visualizations were available for you to view after your consultation. Did you get a chance to view it again on your own time?
2. What were your overall experience with the accompanying visuals used in the breast health specialist's conversations?
3. What were the important take-aways from the visuals shown to you as part of your consultation?
4. How do you understand your chance of getting breast cancer?
    a. How do you think that compares to the average woman?
    b. What about a woman who is at high risk?
5. How are you planning to make decisions about your risk-reducing options?
    a. Have you (or are you planning) to discuss risk reducing options with your provider?
    b. What are the considerations for you in selecting particular risk-reducing options?
6. Did the visuals help to show how your chance of developing breast cancer might change with risk-reducing options?
    a. In your survey, you said that you were interested in doing particular risk-reducing options— what if any steps have you taken?
    b. Can you tell me why you are/are not considering [risk-reduction option: taking chemoprevention, decreasing alcohol, etc.]?
7. Tell me about how your breast cancer risk would be affected by doing [risk-reduction option: taking chemoprevention, decreasing alcohol, etc.]?
    a. How does that differ from what you understood prior to using the tool?
8. Since you had your conversation with the Breast Health Specialist, what has stuck with you the most?
9. Do you have any suggestions to improve the tool?
