# Supplementary material for: Development and pilot of an online, personalized risk assessment tool for a breast cancer precision medicine trial
Source: NPJ Breast Cancer. 2021 Jun 17;7:78. doi: 10.1038/s41523-021-00288-8 (PMC8211836; doi:10.1038/s41523-021-00288-8)
Supplement: Supplementary file 2 — Reporting Summary [file 41523_2021_288_MOESM2_ESM.pdf]

nature research

Corresponding author(s): Laura J. Esserman

Last updated by author(s): 5/6/2021

Reporting Summary

Nature Research wishes to improve the reproducibility of the work that we publish. This form provides structure for consistency and transparency in reporting. For further information on Nature Research policies, see our Editorial Policies and the Editorial Policy Checklist.

Statistics

For all statistical analyses, confirm that the following items are present in the figure legend, table legend, main text, or Methods section.

n/a	Confirmed	
☐	☒	The exact sample size (*n*) for each experimental group/condition, given as a discrete number and unit of measurement
☐	☒	A statement on whether measurements were taken from distinct samples or whether the same sample was measured repeatedly
☒	☐	The statistical test(s) used AND whether they are one- or two-sided<br>*Only common tests should be described solely by name; describe more complex techniques in the Methods section.*
☒	☐	A description of all covariates tested
☒	☐	A description of any assumptions or corrections, such as tests of normality and adjustment for multiple comparisons
☒	☐	A full description of the statistical parameters including central tendency (e.g. means) or other basic estimates (e.g. regression coefficient) AND variation (e.g. standard deviation) or associated estimates of uncertainty (e.g. confidence intervals)
☒	☐	For null hypothesis testing, the test statistic (e.g. *F*, *t*, *r*) with confidence intervals, effect sizes, degrees of freedom and *P* value noted<br>*Give P values as exact values whenever suitable.*
☒	☐	For Bayesian analysis, information on the choice of priors and Markov chain Monte Carlo settings
☒	☐	For hierarchical and complex designs, identification of the appropriate level for tests and full reporting of outcomes
☒	☐	Estimates of effect sizes (e.g. Cohen's *d*, Pearson's *r*), indicating how they were calculated

Our web collection on statistics for biologists contains articles on many of the points above.

Software and code

Policy information about availability of computer code

Data collection	All data was collected on the Salesforce App Cloud.
Data analysis	Readability Test Tool from WebFX was used to determine the Flesch-Kincaid Reading Ease and Grade Level

For manuscripts utilizing custom algorithms or software that are central to the research but not yet described in published literature, software must be made available to editors and reviewers. We strongly encourage code deposition in a community repository (e.g. GitHub). See the Nature Research guidelines for submitting code & software for further information.

Data

Policy information about availability of data

All manuscripts must include a data availability statement. This statement should provide the following information, where applicable:

- Accession codes, unique identifiers, or web links for publicly available datasets
- A list of figures that have associated raw data
- A description of any restrictions on data availability

The personalized risk assessment tool and the data supporting the validity of this tool are available on request from the corresponding author, Laura J. Esserman (Laura.Esserman@UCSF.edu). The tool is available for non-commercial use only and the data are not publicly available since the WISDOM Study portal requires participant authentication and registration.

Field-specific reporting

Please select the one below that is the best fit for your research. If you are not sure, read the appropriate sections before making your selection.

☐ Life sciences ☒ Behavioural & social sciences ☐ Ecological, evolutionary & environmental sciences

For a reference copy of the document with all sections, see nature.com/documents/nr-reporting-summary-flat.pdf

Behavioural & social sciences study design

All studies must disclose on these points even when the disclosure is negative.

Study description	Mixed Methods Study
Research sample	Elevated risk participants in the Women Informed to Screen Depending On Measures of risk (WISDOM) Study were identified in the personalized arm without a breast cancer mutation (namely BRCA1, BCRA2, TP53, PTEN, STK11, CDH1, ATM, PALB2, CHEK2). These women were all classified as elevated risk, in the top 2.5% of Breast Cancer Surveillance Consortium + PRS score by age.
Sampling strategy	A convenience sample of 20 were identified who met criteria above who completed their annual breast health questionnaire within a month of tool's completion (Jan 2019)
Data collection	Quantitative survey and semi-structured interview.
Timing	Tool was developed in 2018-2019. Pilot and survey/interview data collection was conducted in 2019-2020.
Data exclusions	N/A
Non-participation	17 of 20 participants contacted agreed to do a walk-through of the tool with a genetic counselor. 3 participants did not participate in both the quantitative survey and qualitative interview. 4 participants who participated in the quantitative survey did not participate in the qualitative interview.
Randomization	N/A

Reporting for specific materials, systems and methods

We require information from authors about some types of materials, experimental systems and methods used in many studies. Here, indicate whether each material, system or method listed is relevant to your study. If you are not sure if a list item applies to your research, read the appropriate section before selecting a response.

Materials & experimental systems

n/a	Involved in the study	
☒	☐	Antibodies
☒	☐	Eukaryotic cell lines
☒	☐	Palaeontology and archaeology
☒	☐	Animals and other organisms
☐	☒	Human research participants
☐	☒	Clinical data
☒	☐	Dual use research of concern

Methods

n/a	Involved in the study	
☒	☐	ChIP-seq
☒	☐	Flow cytometry
☒	☐	MRI-based neuroimaging

Human research participants

Policy information about studies involving human research participants

Population characteristics	See above.
Recruitment	See above.
Ethics oversight	University of California, San Francisco Institutional Review Board

Note that full information on the approval of the study protocol must also be provided in the manuscript.

Clinical data

Policy information about clinical studies

All manuscripts should comply with the ICMJE guidelines for publication of clinical research and a completed CONSORT checklist must be included with all submissions.

Clinical trial registration	NCT02620852
Study protocol	NCT02620852
Data collection	See above.
Outcomes	Develop a new tool that incorporates personalized risk assessments; create a patient-facing web-based software application; and make the risk assessment tool accessible to a low-literacy audience.<br><br>Pilot study was intended to obtain feedback on the newly developed tool from participants; improve upon the tool before roll-out to a larger study sample; assess the conveyance of personal risk knowledge and motivating preventative actions immediately after consultation with a genetic counselor.
